# Remibrutinib as a novel therapy for refractory granuloma annulare

**DOI:** 10.1016/j.jdcr.2026.09.016

**Published:** 2026-09-16

**Authors:** Tyler C. Chinyere, Ryan Falsey

**Affiliations:** a University of Arizona College of Medicine-Phoenix, Phoenix, Arizona; b East Valley Dermatology Center, Chandler, Arizona

**Keywords:** adult, granuloma annulare, off-label, remibrutinib

## Introduction

Granuloma annulare (GA) is a common, benign, inflammatory, cutaneous disease classified as either localized or disseminated. Although the etiology of GA remains unclear, recent studies have shown its pathogenesis involves elevated T-helper 1 (Th1) and T-helper 2 (Th2) levels, as well as increased activation of Janus kinase (JAK) pathways.^1^^,^^2^ The predominant morphology of GA is erythematous annular plaques distributed over the upper extremities.^3^ Histologically, there is mucin deposition, collagen degeneration, and interstitial histiocytes.^4^

Although standardized treatment guidelines for GA are lacking, first-line therapy includes topical corticosteroids (TCS), intralesional corticosteroid injections, hydroxychloroquine, or phototherapy. Second-line therapies include oral corticosteroids, dapsone, or oral retinoids. For refractory cases, treatment options include biologics, such as tumor necrosis factor-alpha (TNF-α) inhibitors, JAK inhibitors, or methotrexate.^5^

We present a case of refractory, disseminated GA treated with systemic remibrutinib, a newly Food and Drug Administration (FDA)-approved Bruton tyrosine kinase (BTK) inhibitor.

## Case report

A 64-year-old male with a past medical history of basal cell carcinoma, herpes simplex virus, and an 8-year history of biopsy-proven GA presented with a severe outbreak of pruritic, erythematous annular plaques distributed bilaterally across the extremities and trunk. This rash was consistent with prior biopsy-proven GA eruptions, the last occurring 1 year before his visit, which was treated with topical corticosteroids (TCS), 1 to 2 week oral prednisone tapers, and intralesional steroid injections. He had previously failed a 3-month trial of adalimumab with a previous dermatologist. Due to the severity of the current flare, he was started on a 2-week prednisone taper, which significantly improved the rash. However, flares recurred several months after prednisone therapy. Because of concerns about long-term prednisone use, alternative therapies were pursued.

Over the next decade, the patient failed numerous treatments, including hydroxychloroquine (4 months), doxycycline and niacinamide (3 months), narrowband ultraviolet B (NBUVB) (10 months), pentoxifylline (12 months), minocycline (4 months), low dose naltrexone (4 months), allopurinol (2 years and 9 months), methotrexate (2 months), dapsone (3 months), deucravacitinib (3 months), topical ruxolitinib, and various TCS. Hydroxychloroquine, niacinamide, naltrexone, and minocycline had minimal effectiveness. NBUVB decreased flare frequency but did not improve the existing eruption and was discontinued. Both allopurinol and pentoxifylline initially improved disease burden but lost therapeutic response. Methotrexate and dapsone were stopped because of adverse effects and limited effectiveness. During this course, a repeat biopsy of a plaque on the right lateral thigh revealed granulomatous inflammation with mucin deposition in the dermis, elastophagocytosis and dermal elastolysis (confirmed via colloidal and elastic stains, respectively), consistent with GA.

Among prior therapies, oral mycophenolate mofetil produced the best response. The patient achieved 90 percent improvement while receiving up to 3 g daily. However, due to concerns regarding long-term immunosuppression, therapy was transitioned to deucravacitinib, which resulted in recurrent disease flaring.

After deucravacitinib, remibrutinib 25 mg orally twice daily was initiated. After 4 weeks, the patient experienced 75 percent clearance. After 4 months, he achieved 95 percent improvement, with only residual rash around the umbilical region. To date, he remains stable on remibrutinib without reported adverse effects (Figs 1 and 2).

**Fig 1:**
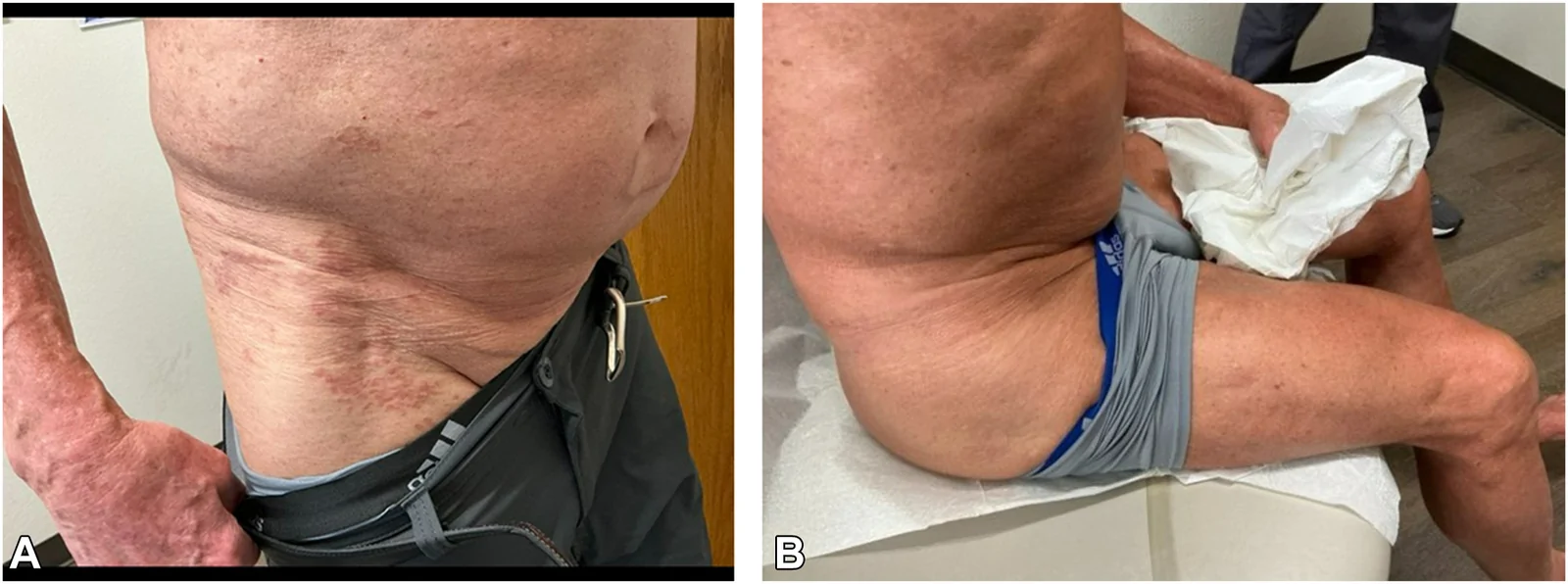
**A,** Granuloma annulare on right hip prior to remibrutinib. **B,** Resolution of granuloma annulare after remibrutinib.

**Fig 2:**
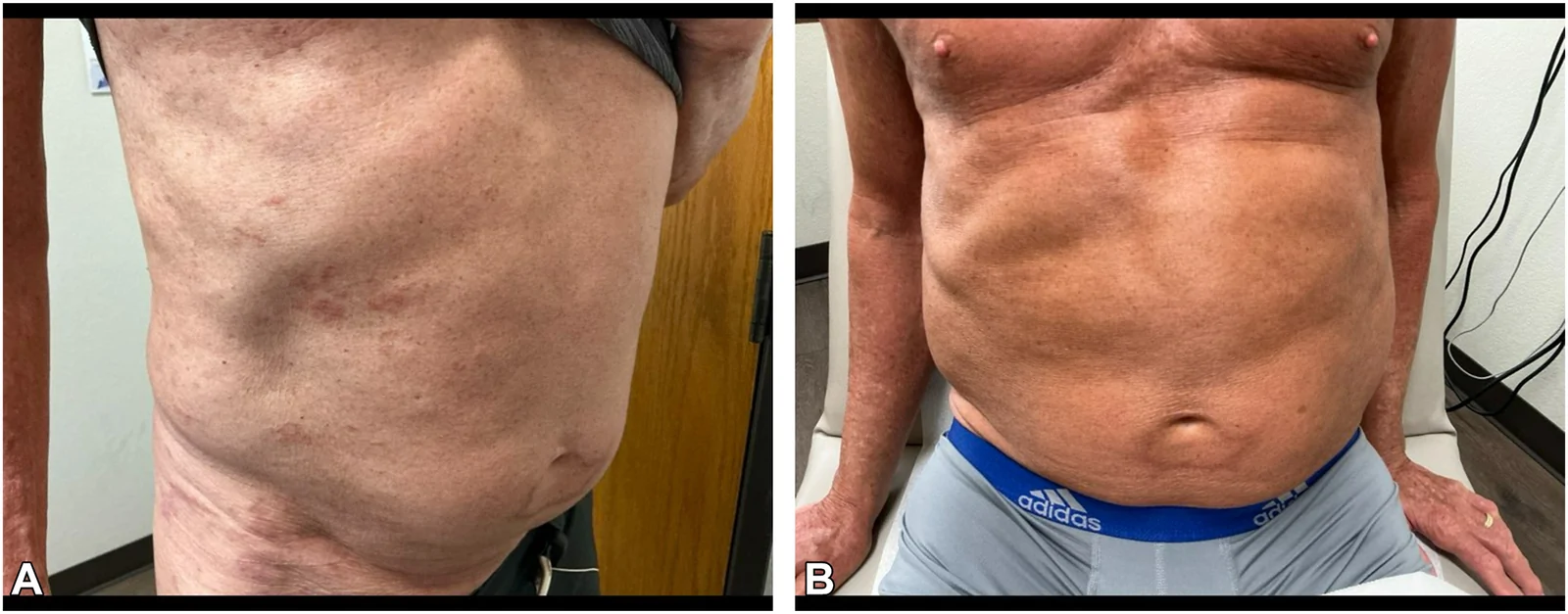
**A,** Granuloma annulare on abdomen prior to remibrutinib. **B,** Resolution of granuloma annulare after remibrutinib.

## Discussion

Remibrutinib is a highly selective, irreversible immunomodulator approved by the FDA in 2025 for chronic spontaneous urticaria (CSU). While BTK inhibitors are most classically known for impeding B-cell receptor signaling in hematologic conditions, BTK is also robustly expressed in cells of myeloid lineage, including macrophages, dendritic cells, and mast cells.^6^^,^^7^ Within the innate immune system, BTK serves as a critical downstream effector for Toll-like receptors (TLRs) and Fc receptors.^7^

In the context of GA, aberrant Th1 signaling leads to elevated interferon-gamma, which primes macrophages for activation and aggregation.^1^^,^^2^ BTK activation within these localized histiocytes drives the production of pro-inflammatory cytokines (such as TNF-α, IL-1β, and IL-6) and supports phagocytosis and inflammasome signaling, all of which are essential for maintaining granulomatous inflammation.^7^ By selectively inhibiting BTK, remibrutinib disrupts this myeloid activation cascade. This directly blunts the histiocytic hyperactivation and cytokine release necessary for granuloma formation and persistence. This targeted dampening of the innate immune response likely accounts for the rapid clinical improvement observed in our patient.

This case highlights the rapid efficacy of remibrutinib. After 4 weeks, our patient achieved 75 percent improvement. This clearance exceeded his best prior non-steroidal response to mycophenolate mofetil, which took 8 months to achieve 50 percent improvement and over 1 year to produce 90% clearance. Remibrutinib may also be suitable for long-term maintenance. While broad immunosuppressants like mycophenolate mofetil and prednisone carry cumulative long-term risks, remibrutinib’s targeted immunomodulatory mechanism may offer a safer profile for extended use.

Another advantage of remibrutinib in this case was favorable tolerability. While our patient experienced prohibitive adverse effects from methotrexate and dapsone, he reported no side effects with standard-dosed remibrutinib. Common side effects in broader populations can include nasopharyngitis, headaches, bleeding, and abdominal pain.^6^

Given the novelty of remibrutinib, data regarding its off-label use remains limited. This case suggests that BTK inhibition may be a useful, targeted approach for refractory GA by modulating macrophage-driven granulomatous inflammation. Further studies are needed to evaluate the safety, durability, and efficacy of remibrutinib in GA and related granulomatous diseases.

### Declaration of generative AI and AI-assisted technologies in the writing process

None.

## Conflicts of interest

None disclosed.
